# Correlation between perioperative dexmedetomidine administration and postoperative acute kidney injury in hypertensive patients undergoing non-cardiac surgery

**DOI:** 10.3389/fphar.2023.1143176

**Published:** 2023-03-29

**Authors:** Bo Li, Minghua Chen, Youjie Zeng, Siwan Luo

**Affiliations:** ^1^ Operation Center, Third Xiangya Hospital, Central South University, Changsha, China; ^2^ Department of Anesthesiology, Third Xiangya Hospital, Central South University, Changsha, China

**Keywords:** dexmedetomidine, postoperative acute kidney injury, hypertensive, non-cardiac surgery, incidence risk, renal function, AKI stage

## Abstract

**Background:** Previous studies have suggested that dexmedetomidine may have a protective effect on renal function. However, it is currently unclear whether perioperative dexmedetomidine administration is associated with postoperative acute kidney injury (AKI) incidence risk in hypertensive patients undergoing non-cardiac surgery.

**Methods:** This investigation was a retrospective cohort study. Hypertensive patients undergoing non-cardiac surgery in Third Xiangya Hospital of Central South University from June 2018 to December 2019 were included. The relevant data were extracted through electronic cases. The univariable analysis identified demographic, preoperative laboratory, and intraoperative factors associated with acute kidney injury. Multivariable stepwise logistic regression was used to assess the association between perioperative dexmedetomidine administration and postoperative acute kidney injury after adjusting for interference factors. In addition, we further performed sensitivity analyses in four subgroups to further validate the robustness of the results.

**Results:** A total of 5769 patients were included in this study, with a 7.66% incidence of postoperative acute kidney injury. The incidence of postoperative acute kidney injury was lower in the dexmedetomidine-administered group than in the control group (4.12% vs. 8.06%, *p* < 0.001). In the multivariable stepwise logistic regression analysis, perioperative dexmedetomidine administration significantly reduced the risk of postoperative acute kidney injury after adjusting for interference factors [odds ratio (OR) = 0.56, 95% confidence interval (CI): 0.36–0.87, *p* = 0.010]. In addition, sensitivity analysis in four subgroups indicated parallel findings: i) eGRF <90 mL/min·1.73/m^2^ subgroup (OR = 0.40, 95% CI: 0.19–0.84, *p* = 0.016), ii) intraoperative blood loss <1000 mL subgroup (OR = 0.58, 95% CI: 0.36–0.94, *p* = 0.025), iii) non-diabetes subgroup (OR = 0.51, 95% CI: 0.29–0.89, *p* = 0.018), and iv) older subgroup (OR = 0.55, 95% CI: 0.32–0.93, *p* = 0.027).

**Conclusion:** In conclusion, our study suggests that perioperative dexmedetomidine administration is associated with lower risk and less severity of postoperative acute kidney injury in hypertensive individuals undergoing non-cardiac surgery. Therefore, future large-scale RCT studies are necessary to validate this benefit.

## 1 Introduction

Postoperative acute kidney injury (AKI) is a common organ injury after surgery, leading to increased postoperative complications (Gumbert et al., 2020). In addition, postoperative AKI also deteriorates other organ functions (Schrier and Wang, 2004). Furthermore, subclinical AKI is associated with increased postoperative mortality (Park et al., 2012). Hypertensive patients had lower renal function and a higher incidence of postoperative AKI (Kim et al., 2014; Brouwers et al., 2021). Hypertensive patients are at heightened risk for acute kidney injury (AKI) after surgery due to several factors, including increased susceptibility to renal underperfusion (Zappitelli et al., 2020), the presence of comorbidities such as chronic kidney disease and diabetes (Prowle et al., 2021), and a higher likelihood of experiencing intraoperative hypotension (Mathis et al., 2020). Given these risks, strategies to prevent postoperative AKI in hypertensive patients are urgently needed.

Preventing postoperative AKI requires preoperative strategies that target high-risk patients and optimize their clinical status both preoperatively and intraoperatively (Gameiro et al., 2020). Specifically, interventions should focus on ensuring adequate organ perfusion and oxygenation during surgery while avoiding medications that inhibit the renin-angiotensin-aldosterone system and non-steroidal anti-inflammatory drugs (Park, 2017; Gameiro et al., 2020). Additionally, perioperative hyperglycemia (glucose levels >180 mg/dL) should be avoided (Prowle et al., 2021). In addition to these recognized modifiable protective factors, multiple protective factors for postoperative AKI remain to be explored. Dexmedetomidine is a highly selective adrenoceptor agonist that inhibits norepinephrine release and produces pharmacological effects such as sedation, analgesia, and anti-anxiety (Liu et al., 2021). Due to its analgesic and sedative qualities, lack of respiratory inhibition, and low incidence of postoperative nausea and vomiting, it is frequently used in general anesthesia and ICU sedation (Lee, 2019). Previous studies have demonstrated that dexmedetomidine can protect renal function from ischemia-reperfusion injury and lessen the incidence and severity of AKI by inhibiting the inflammatory response, apoptosis, and oxidative stress (Si et al., 2013).

Nevertheless, few studies have identified the relationship between perioperative dexmedetomidine administration and postoperative AKI in hypertensive patients undergoing non-cardiac surgery. Our study aimed to investigate whether the perioperative use of dexmedetomidine was associated with a reduced postoperative AKI risk by implementing a single-center retrospective study.

## 2 Materials and methods

### 2.1 Study design

The study included hypertensive patients undergoing non-cardiac surgery at The Third Xiangya Hospital of Central South University from June 2018 to December 2019. Hypertensive patients were screened according to the ICD 10 in the electronic medical record (primary hypertension: I10) (Beckman, 2014). Inclusion criteria: adult (>18 years old) hypertensive patients undergoing non-cardiac surgery. Patients undergoing cardiac surgery were excluded due to their higher incidence of postoperative AKI than patients undergoing non-cardiac surgery (Nadim et al., 2018). Exclusion criteria: i) chronic renal insufficiency [estimated glomerular filtration rate (eGFR) < 60 mL/min/1.73 m^2^, ≥ 3 months], since eGFR <60 mL/min/1.73 m^2^ is one of the indicators of chronic kidney disease (Vestergaard et al., 2021); ii) American Society of Anesthesiologists (ASA) grade V and above, due to their extremely dismal physical base condition (Doyle et al., 2021); iii) patients undergoing local anesthesia or regional block anesthesia; iv) patients undergoing kidney transplantation; and v) lacking serum creatinine data. The study was approved by the Institutional Review Board of the Third Xiangya Hospital of Central South University (registration number: Fast I 22055).

### 2.2 Primary outcome definition

According to the Kidney Disease Improving Global Outcomes (KDIGO), the definition of postoperative AKI was as follows: an increase in serum creatinine level of 0.3 mg/dL within 48 h or an increase in serum creatinine level of 1.5 times the preoperative baseline level within 7 days after surgery (Kellum et al., 2012). We did not choose urine volume as one of the criteria for diagnosing postoperative AKI since postoperative urine volume was not counted or inaccurately counted in the ward. Overall, the AKI incidence and AKI severity (AKI stages) within 7 days after surgery were the primary outcome indicators in our study.

### 2.3 Data collection

The following information was collected through electronic information system records: i) epidemiological data: patients’ age, gender, and body mass index (BMI); ii) personal medical history, including preoperative comorbidities and personal medication history; iii) laboratory data, including serum creatinine and glomerular filtration rate (eGFR, calculated using the CKD epidemiological formula); iv) intraoperative data, including operative duration, anesthesia method, ASA grade, the volume of fluid and bleeding, intraoperative red blood cell transfusion, blood loss, intraoperative minimum mean arterial pressure (MAP), other intraoperative sedative or analgesic medications, and vasoactive drugs; v) incidence and severity of postoperative AKI.

### 2.4 Statistical analysis

Statistical analysis of the collected data was performed using SAS V.9.4 software (SAS Institute) and CRAN R (V.3.4.3). Missing data for covariates (including BMI and eGFR) were processed using multiple compensation models. Normally distributed continuous variables were summarized as mean ± standard deviation (SD), while non-normally distributed continuous variables were described using the median and quartiles (the normality test was performed by Kolmogorov-Smirnov test). In addition, categorical variables were expressed as percentages. Continuous variables were compared between groups using the Wilcoxon rank sum test, and categorical variables were compared using the χ2 test or Fisher’s exact probability method. Univariate logistic regression analysis was used to identify epidemiological, preoperative laboratory, and intraoperative factors significantly associated with postoperative AKI incidence. Variables (*p* < 0.1) in univariate logistics regression and variables mentioned in the previous research that may be related to AKI were considered potential confounding factors. In multivariable regression models, covariates were adjusted for potential confounding factors. In addition, the multivariable model’s goodness-of-fit was assessed by the Calibration Curve and Hosmer-Lemeshow test. Finally, we further performed sensitivity analyses in subgroups to further validate the robustness of the results. Sensitivity tests were performed on four subgroups: i) eGFR <90 mL/min/1.73 m^2^, ii) intraoperative blood loss <1000 ml, iii) non-diabetes, and iv) older (age ≥60 years). These subgroups were chosen since eGFR (Shen et al., 2022), diabetes mellitus (Patschan and Müller, 2016), intraoperative blood loss (Ida et al., 2020), and advanced age (Abdel-Kader and Palevsky, 2009) were all associated with AKI risk. We selected two AKI high-risk subgroups and two AKI low-risk subgroups for analysis to demonstrate the stability of the relationship between dexmedetomidine and postoperative AKI. Results for categorical variables were expressed as odds ratio (OR) or β value with 95% confidence intervals (CI). A statistically significant difference was indicated by *p* < 0.05*.*

## 3 Results

### 3.1 Clinical features of dexmedetomidine group and non-administration group

**FIGURE 1:**
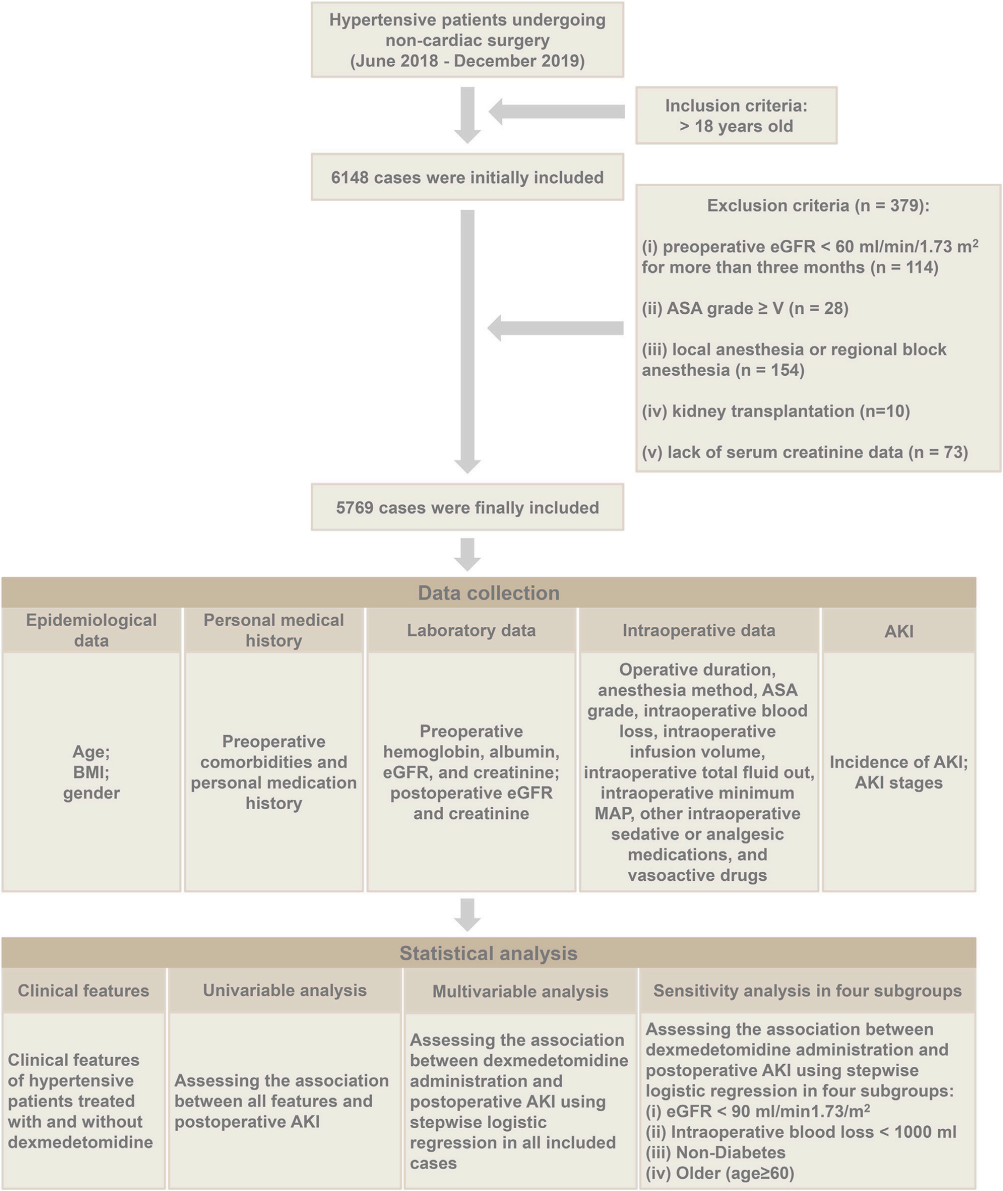
The general flow chart of this study.

The general flow chart of this study is shown in Figure 1. Six thousand one hundred forty-eight patients met the inclusion criteria, 379 cases were excluded, and 5769 cases were involved in the statistical analysis. The causes of exclusion were as follows: preoperative chronic renal insufficiency in 114 cases, ASA grade V and above in 28 cases, local anesthesia or regional block anesthesia in 154 cases, kidney transplantation in 10 cases, and lacking serum creatinine data in 73 cases. All clinical data for the 5769 patients was obtained by reviewing our hospital’s HIS and anesthesia systems retrospectively.

The statistical analysis of 5769 patients’ data revealed that 442 (7.66%) suffered from postoperative AKI. Among the patients, 583 (10.11%) received dexmedetomidine administration; of those, 24 developed postoperative AKI. In contrast, among the 5186 patients who did not receive dexmedetomidine, 418 suffered from postoperative AKI. The incidence of postoperative AKI was significantly lower in the dexmedetomidine group (4.12%) than in the non-administration group (8.06%). Further analysis revealed a statistically significant difference in the AKI severity (AKI stages) between the two groups (*p* = 0.007) (Table 1). In addition, the number of each surgery type included in this study is shown in Supplementary Table S1.

**TABLE 1:** Clinical features of hypertensive patients treated with and without dexmedetomidine.

Clinical features	Without dexmedetomidine (n = 5186)	With dexmedetomidine (n = 583)	*P*
AKI n (%)	418 (8.06%)	24 (4.12%)	<0.001
AKI stages n (%)			0.007
0	4768 (91.94%)	559 (95.88%)	
1	389 (7.50%)	22 (3.77%)	
2	22 (0.42%)	2 (0.34%)	
3	7 (0.13%)	0 (0.00%)	
Age (years)	62.42 ± 11.81	62.45 ± 11.92	0.578
BMI (kg/m^2^)	24.27 ± 4.02	24.63 ± 4.19	0.060
Male n (%)	2904 (56.00%)	313 (53.69%)	0.287
Diabetes n (%)	1337 (25.78%)	131 (22.47%)	0.082
Alcohol consumption n (%)	312 (6.02%)	21 (3.60%)	0.018
Smoking n (%)	467 (9.01%)	27 (4.63%)	<0.001
Diuretics n (%)	217 (4.18%)	11 (1.89%)	0.007
ACEI n (%)	233 (4.49%)	10 (1.72%)	0.002
CCB n (%)	12 (0.23%)	1 (0.17%)	0.773
NSAIDs n (%)	885 (17.07%)	85 (14.58%)	0.128
Preoperative hemoglobin (g/L)	121.83 ± 22.38	125.14 ± 20.97	<0.001
Preoperative albumin (g/L)	39.14 ± 5.48	39.59 ± 4.94	0.098
Preoperative eGFR (mL/min/1.73 m^2^)	89.70 (71.67–101.10)	92.54 (81.19–103.16)	<0.001
Preoperative creatinine (mmol/L)	69.00 (57.00–87.00)	67.00 (54.50–80.00)	<0.001
Intraoperative blood loss (mL)	150.00 (50.00–350.00)	200.00 (50.00–400.00)	0.323
Intraoperative infusion volume (mL)	2100.00 (1500.00–3100.00)	2000.00 (1500.00–3000.00)	0.288
Intraoperative total fluid out (mL)	600.00 (300.00–1000.00)	600.00 (350.00–1000.00)	0.096
Emergency n (%)	719 (13.86%)	67 (11.49%)	0.113
Operation duration (min)	150.00 (105.00–220.00)	167.00 (115.00–247.50)	<0.001
Intraoperative sufentanil consumption (μg)	40.00 (35.00–50.00)	30.00 (20.00–50.00)	<0.001
Intraoperative midazolam consumption (mg)	2.00 (2.00–3.00)	2.00 (2.00–3.00)	0.109
Intraoperative propofol consumption (mg)	550.00 (438.00–740.00)	550.00 (400.00–700.00)	0.114
Intraoperative sevoflurane consumption (mL)	20.00 (10.00–30.00)	30.00 (20.00–40.00)	0.028
Intraoperative minimum MAP (mmHg)	65.00 (55.00–73.00)	61.00 (45.00–73.00)	<0.001
Intraoperative norepinephrine use n (%)	686 (13.23%)	77 (13.21%)	0.989
General anesthesia n (%)	4481 (86.41%)	426 (73.07%)	<0.001
ASA grade n (%)			0.003
1	56 (1.08%)	7 (1.20%)	
2	1878 (36.21%)	233 (39.97%)	
3	2736 (52.76%)	312 (53.52%)	
4	516 (9.95%)	31 (5.32%)	

*p*-values: if continuous variables, derived by Wilcoxon rank sum test; if count variables had a theoretical number <10, derived by Fisher exact probability test; use of diuretics, ACEI, CCB, *etc.*, was defined as the use of the drug within 1 week before surgery.

AKI, stages: outcome of postoperative AKI, was divided into four groups: stage 0, no AKI; stage 1, AKI, grade 1; stage 2, AKI, grade 2 and stage 3; AKI, grade 3.

Abbreviations: AKI, acute kidney injury; BMI, body mass index; ACEI, angiotensin-converting enzyme inhibitor; CCB, calcium-channel blockers; NSAIDs, non-steroidal anti-inflammatory drugs; eGFR, estimated glomerular filtration rate; MAP, mean arterial pressure; ASA, american society of anesthesiologist.

No statistically significant differences were identified in age, BMI, gender composition, diabetes, preoperative use of calcium-channel blockers (CCB), preoperative use of non-steroidal anti-inflammatory drugs (NSAIDs), preoperative albumin, intraoperative blood loss, intraoperative infusion volume, intraoperative total fluid out, intraoperative midazolam consumption, intraoperative propofol consumption, and intraoperative norepinephrine use between the two groups. In contrast, there were statistically significant differences in alcohol consumption, smoking, preoperative use of diuretics, preoperative use of angiotensin-converting enzyme inhibitor (ACEI), preoperative hemoglobin, preoperative eGFR, preoperative creatinine, operation duration, intraoperative sufentanil consumption, intraoperative sevoflurane consumption, intraoperative minimum MAP, general anesthesia ratio, and ASA grade between the two groups (Table 1).

### 3.2 Identification of variables associated with postoperative AKI by univariate regression analysis

In the Univariate regression analysis, age, diabetes, preoperative use of diuretics, preoperative use of ACEI, preoperative use of CCB, increased preoperative creatinine, increased intraoperative blood loss, intraoperative norepinephrine use, and ASA grade Ⅳ were independently associated with an increased risk of postoperative AKI. In addition, dexmedetomidine administration, BMI, male, preoperative use of NSAIDs, preoperative hemoglobin, preoperative albumin, preoperative eGFR, increased intraoperative infusion volume, increased intraoperative total fluid out, and intraoperative minimum MAP were independently associated with a reduced risk of postoperative AKI. In contrast, alcohol consumption, smoking, intraoperative sufentanil consumption, intraoperative midazolam consumption, intraoperative propofol consumption, intraoperative sevoflurane consumption, general anesthesia ratio, and ASA grade I-III were not associated with the development of postoperative AKI (Table 2).

**TABLE 2:** Univariable analysis of AKI.

Variables	AKI	
	OR (95% CI)	*p*-value
Dexmedetomidine n (%)	0.49 (0.32, 0.75)	<0.001
Age (years)	1.01 (1.00, 1.02)	0.016
BMI (kg/m^2^)	0.94 (0.92, 0.97)	<0.001
Male n (%)	0.66 (0.54, 0.80)	<0.001
Diabetes n (%)	1.71 (1.39, 2.09)	<0.001
Alcohol consumption n (%)	0.93 (0.61, 1.43)	0.748
Smoking n (%)	1.20 (0.86, 1.66)	0.277
Diuretics n (%)	3.48 (2.49, 4.87)	<0.001
ACEI n (%)	1.82 (1.23, 2.69)	0.003
CCB n (%)	5.40 (1.66, 17.59)	0.005
NSAIDs n (%)	0.53 (0.38, 0.72)	<0.001
Preoperative hemoglobin (g/L)	0.98 (0.97, 0.98)	<0.001
Preoperative albumin (g/L)	0.90 (0.89, 0.92)	<0.001
Preoperative eGFR (mL/min/1.73 m^2^)	0.97 (0.97, 0.98)	<0.001
Preoperative creatinine (mmol/L)		
Low	References	
Middle	0.71 (0.54, 0.94)	0.017
High	1.97 (1.56, 2.49)	<0.001
Intraoperative blood loss (mL)		
Low	References	
Middle	0.89 (0.69, 1.15)	0.375
High	1.27 (1.00, 1.61)	0.046
Intraoperative infusion volume (mL)		
Low	References	
Middle	0.56 (0.44, 0.71)	<0.001
High	0.55 (0.44, 0.69)	<0.001
Intraoperative total fluid out (mL)		
Low	References	
Middle	0.55 (0.44, 0.70)	<0.001
High	0.46 (0.36, 0.58)	<0.001
Operation duration (min)		
Low	References	
Middle	0.70 (0.55, 0.89)	0.003
High	0.74 (0.59, 0.94)	0.012
Intraoperative sufentanil consumption (μg)	1.01 (0.98, 1.04)	0.716
Intraoperative midazolam consumption (mg)	0.62 (0.23, 1.64)	0.333
Intraoperative propofol consumption (mg)	1.00 (1.00, 1.00)	0.291
Intraoperative sevoflurane consumption (mL)	1.01 (0.98, 1.03)	0.656
Intraoperative minimum MAP (mmHg)	0.99 (0.99, 1.00)	0.029
Intraoperative norepinephrine use n (%)	2.57 (2.05, 3.23)	<0.001
General anesthesia n (%)	0.93 (0.71, 1.21)	0.583
Emergency n (%)	0.46 (0.28, 0.86)	0.030
ASA grade n (%)		
1	References	
2	1.15 (0.28, 4.81)	0.843
3	2.70 (0.66,11.11)	0.169
4	8.12 (1.96, 33.70)	0.004

ACEI, CCB, *etc.*, was defined as the use of the drug within 1 week before surgery.

Abbreviations: AKI, acute kidney injury; BMI, body mass index; ACEI, angiotensin-converting enzyme inhibitor; CCB, calcium-channel blockers; NSAIDs, non-steroidal anti-inflammatory drugs; eGFR, estimated glomerular filtration rate; MAP, mean arterial pressure; ASA, american society of anesthesiologist.

### 3.3 Assessment of the association between perioperative dexmedetomidine administration and postoperative AKI risk by multivariable stepwise logistic regression

A risk-adjusted model was performed using stepwise logistic regression. After adjusting for the confounders (BMI + gender + age + CCB + ACEI + diuretics + diabetes + NSAIDs + preoperative hemoglobin + preoperative albumin + operation duration + intraoperative blood loss + intraoperative infusion volume + intraoperative total fluid out + ASA grade + intraoperative minimum MAP + mode of anesthesia), perioperative dexmedetomidine administration remained significantly associated with reduced postoperative AKI incidence (OR = 0.56, 95% CI: 0.36–0.87, *p* = 0.010) and AKI stages (β= −0.03, 95% CI: −0.06 to −0.01), *p* = 0.010) (Table 3). In addition, the calibration curve and Hosmer-Lemeshow test showed that the model fitted well (*p* = 0.372) (Supplementary Figure S1).

**TABLE 3:** OR or β value of postoperative AKI or AKI stages associated with perioperative dexmedetomidine administration.

	Model 1		Model 2		Model 3	
	OR/β (95%CI)	*P*	OR/β (95%CI)	*P*	OR/β (95%CI)	*P*
AKI	0.49 (0.32, 0.75)	<0.001	0.55 (0.36, 0.85)	0.006	0.56 (0.36, 0.87)	0.010
AKI stages	−0.04 (−0.07, −0.05)	<0.001	−0.03 (−0.06, −0.01)	0.011	−0.03 (−0.06, −0.01)	0.010

Model 1: non-adjusted.

Model 2: adjusted for BMI, gender, age, CCB, ACEI, diuretics, diabetes; NSAIDs, preoperative hemoglobin, preoperative albumin, and operation duration.

Model 3: model 2 plus intraoperative blood loss, intraoperative infusion volume, intraoperative total fluid out, ASA, grade, intraoperative minimum MAP, and mode of anesthesia.

Abbreviations: OR, odds ratio; CI, confidence interval; AKI, acute kidney injury.

### 3.4 Sensitivity analysis in four subgroups

Furthermore, we examined whether dexmedetomidine administration was associated with postoperative AKI risk in four subgroups [i) eGFR <90 mL/min1.73/m^2^, ii) intraoperative blood loss <1000 mL, iii) non-diabetes, and iv) older (age ≥60 years)]. The results suggested that perioperative use of dexmedetomidine significantly reduced the incidence of postoperative AKI in these four subgroups, and the results remained statistically significant after adjusting for relevant covariates (OR < 1 and *p* < 0.05) (Table 4).

**TABLE 4:** Sensitivity analysis of the association between postoperative AKI and perioperative dexmedetomidine administration in four subgroups.

Subgroups	Model1		Model2		Model3	
	OR (95%CI)	*P*	OR (95%CI)	*P*	OR (95%CI)	*P*
eGFR <90 mL/min1.73/m^2^	0.45 (0.22, 0.93)	0.030	0.44 (0.21, 0.92)	0.029	0.40 (0.19, 0.84)	0.016
Intraoperative blood loss <1000 mL	0.46 (0.29, 0.73)	0.001	0.54 (0.34, 0.86)	0.009	0.58 (0.36, 0.94)	0.025
Non-diabetes	0.42 (0.25, 0.73)	0.002	0.47 (0.27, 0.81)	0.007	0.51 (0.29, 0.89)	0.018
Older (age≥60)	0.48 (0.29, 0.81)	0.006	0.55 (0.33, 0.93)	0.025	0.55 (0.32, 0.93)	0.027

Model 1: non-adjusted.

Model 2: adjusted for BMI, gender, age, CCB, ACEI, diuretics, diabetes; NSAIDs, preoperative hemoglobin, preoperative albumin, and operation duration.

Model 3: model 2 plus intraoperative blood loss, intraoperative infusion volume, intraoperative total fluid out, ASA, grade, intraoperative minimum MAP, and mode of anesthesia.

Abbreviations: OR, odds ratio; CI, confidence interval; eGFR, estimated glomerular filtration rate.

## 4 Discussion

With increased professional training and advances in monitoring and treatment techniques, the postoperative rehabilitation index of patients is gradually improving, resulting in fewer postoperative complications, re-admissions, and hospitalization costs (Ljungqvist et al., 2017). However, postoperative organ function impairment is always present, especially acute kidney injury (AKI), with an incidence of 20%–40% in high-risk patients (Bauerle et al., 2011). We performed a retrospective analysis, including 5769 patients with hypertension who underwent non-cardiac surgery. Focusing on hypertensive patients for research has the following advantages: i) hypertension is a prevalent condition; ii) hypertensive patients are at an increased risk of developing postoperative AKI; iii) there are currently no drugs available to effectively prevent AKI. However, certain limitations must be acknowledged, including the fact that the duration of hypertension is not known and that there may be variability in the medications used to treat hypertension, potentially impacting the analysis. In our study, the incidence of AKI was 7.66%, similar to the findings recently reported by Kork et al. (a retrospective study of 39369 surgical patients using KDIGO diagnostic criteria, the incidence of AKI was 6%) (Kork et al., 2015).

A recent meta-analysis showed that perioperative dexmedetomidine administration was not associated with postoperative AKI risk (Hu et al., 2022); however, the study did not specifically focus on hypertensive patients who were at a higher postoperative AKI risk. In contrast, our study examined hypertensive patients and identified that dexmedetomidine administration was associated with a reduced postoperative AKI risk in hypertensive patients undergoing non-cardiac surgery. Multivariable stepwise logistic regression analyses showed that perioperative dexmedetomidine administration remained associated with reduced postoperative AKI risk and its severity after adjusting for relevant covariates. In addition, sensitivity analyses in four subgroups [i) eGRF <90 mL/min·1.73/m^2^ subgroup, ii) intraoperative blood loss <1000 mL subgroup, iii) non-diabetes subgroup, and iv) older subgroup] all suggested that perioperative dexmedetomidine administration was significantly associated with reduced postoperative AKI risk. The consistency of these results gives us confidence that perioperative dexmedetomidine administration is significantly associated with a reduced risk of postoperative AKI in hypertensive patients undergoing non-cardiac surgery.

Although not fully established, current studies suggest several mechanisms to explain the potential reduced postoperative AKI with perioperative dexmedetomidine administrations. First, the benefit may be attributed to the modulation of sympathetic tension by dexmedetomidine, which optimizes renal function (Bellomo et al., 2012). Overactivation of the sympathetic nerve induced by surgical stress will increase the release of catecholamine, leading to hemodynamic instability and renal artery vasoconstriction, which has certain damaging effects on renal function (Meersch et al., 2017). In contrast, perioperative dexmedetomidine administration is thought to contribute to hemodynamic stability (Kulka et al., 1996) and attenuate the effects of renal ischemia/perfusion injury by modulating sympathetic tension (Gu et al., 2011). In addition, activation of α2 adrenergic receptors in the renal vascular system and renal tubules also inhibits renin secretion. Dexmedetomidine may exert a direct vasodilatory effect by inducing nitric oxide-dependent vasodilation in endothelial cells by activating α-2-adrenoceptor (Gu et al., 2011). Moreover, dexmedetomidine may also improve renal function by inhibiting the inflammatory response, which has been confirmed in animal studies (Taoda et al., 2001; Gu et al., 2011; Liang et al., 2017). Furthermore, dexmedetomidine has been shown to reduce lipopolysaccharide-induced sepsis-related acute kidney injury by activating the α7 nicotinic acetylcholine receptor, thereby reducing inflammation and apoptosis (Kang et al., 2018).

Previous studies have revealed an association between perioperative dexmedetomidine administration and postoperative AKI risk in patients undergoing cardiovascular surgery. Dexmedetomidine had a renal protective effect in aortic dissection stent implantation (Shan et al., 2021). However, some studies have shown that dexmedetomidine has an effect on urine volume in patients undergoing coronary artery bypass grafting but has no effect on postoperative creatinine clearance (Leino et al., 2011). Overall, most of the current studies suggest that dexmedetomidine has a certain protective effect on the kidney. We demonstrated for the first time that perioperative dexmedetomidine administration is also associated with reduced postoperative AKI risk in hypertensive patients undergoing non-cardiac surgery.

Nevertheless, several limitations existed in our study. First, because this investigation was a retrospective study, only serum creatinine and eGFR were included as indicators of renal function, and no dexmedetomidine-related adverse events were recorded. Second, this study only focused on short-term postoperative alterations in renal function. Third, no catecholamine or inflammatory marker levels were available. Fourth, although we included multiple covariates for correction in our multivariable analysis, the preference of anesthesiologists to use dexmedetomidine in relatively healthy patients may introduce unexpected potential confounding factors. Therefore the causal relationship still needs to be verified by RCT. Finally, no stratified study of dexmedetomidine dose was performed. These will require investigation in the future.

## 5 Conclusion

In conclusion, our study suggests that perioperative dexmedetomidine administration is associated with lower risk and less severity of postoperative AKI in hypertensive individuals undergoing non-cardiac surgery. Therefore, future large-scale RCT studies are necessary to validate this benefit.

## Data Availability

The original contributions presented in the study are included in the article/Supplementary Material, further inquiries can be directed to the corresponding author.

## References

[B1] Abdel-Kader K. Palevsky P. M. (2009). Acute kidney injury in the elderly. Clin. Geriatr. Med. 25 (3), 331–358. 10.1016/j.cger.2009.04.001 19765485 PMC2748997

[B2] Bauerle J. D. Grenz A. Kim J. H. Lee H. T. Eltzschig H. K. (2011). Adenosine generation and signaling during acute kidney injury. J. Am. Soc. Nephrol. 22 (1), 14–20. 10.1681/ASN.2009121217 21209250

[B3] Beckman K. D. (2014). How to document and code for hypertensive diseases in ICD-10. Fam. Pract. Manag. 21 (2), 5–9. 24693838

[B4] Bellomo R. Kellum J. A. Ronco C. (2012). Acute kidney injury. Lancet 380 (9843), 756–766. 10.1016/S0140-6736(11)61454-2 22617274

[B5] Brouwers S. Sudano I. Kokubo Y. Sulaica E. M. (2021). Arterial hypertension. Lancet 398 (10296), 249–261. 10.1016/S0140-6736(21)00221-X 34019821

[B6] Doyle D. J. Goyal A. Bansal P. Garmon E. H. (2021). American society of anesthesiologists classification. Treasure Island: StatPearls Publishing. 28722969

[B7] Gameiro J. Fonseca J. A. Marques F. Lopes J. A. (2020). Management of acute kidney injury following major abdominal surgery: A contemporary Review. J. Clin. Med. 9 (8), 2679. 10.3390/jcm9082679 32824854 PMC7463962

[B8] Gu J. Sun P. Zhao H. Watts H. R. Sanders R. D. Terrando N. (2011). Dexmedetomidine provides renoprotection against ischemia-reperfusion injury in mice. Crit. Care 15 (3), R153. 10.1186/cc10283 21702944 PMC3219027

[B9] Gumbert S. D. Kork F. Jackson M. L. Vanga N. Ghebremichael S. J. Wang C. Y. (2020). Perioperative acute kidney injury. Anesthesiology 132 (1), 180–204. 10.1097/ALN.0000000000002968 31687986 PMC10924686

[B10] Hu B. Tian T. Li X. Liu W. Chen Y. Jiang T. (2022). Perioperative dexmedetomidine administration does not reduce the risk of acute kidney injury after non-cardiac surgery: A meta-analysis. Chin. Med. J. Engl. 135 (23), 2798–2804. 10.1097/cm9.0000000000002408 36728946 PMC9944691

[B11] Ida M. Sumida M. Naito Y. Tachiiri Y. Kawaguchi M. (2020). Impact of intraoperative hypotension and blood loss on acute kidney injury after pancreas surgery. Braz. J. Anesthesiol. Engl. Ed. 70 (4), 343–348. 10.1016/j.bjan.2020.04.011 PMC9373639 32739201

[B12] Kang K. Gao Y. Wang S. C. Liu H. T. Kong W. L. Zhang X. (2018). Dexmedetomidine protects against lipopolysaccharide-induced sepsis-associated acute kidney injury via an α7 nAChR-dependent pathway. Biomed. Pharmacother. 106, 210–216. 10.1016/j.biopha.2018.06.059 29960167

[B13] Kellum J. A. Lameire N. Aspelin P. Barsoum R. S. Burdmann E. A. Goldstein S. L. (2012). Kidney disease: Improving global outcomes (KDIGO) acute kidney injury work group. KDIGO clinical practice guideline for acute kidney injury. Kidney Int. Suppl. 2 (1), 1–138.

[B14] Kim M. Brady J. E. Li G. (2014). Variations in the risk of acute kidney injury across intraabdominal surgery procedures. Anesth. Analg. 119 (5), 1121–1132. 10.1213/ANE.0000000000000425 25191972

[B15] Kork F. Balzer F. Spies C. D. Wernecke K. D. Ginde A. A. Jankowski J. (2015). Minor postoperative increases of creatinine are associated with higher mortality and longer hospital length of stay in surgical patients. Anesthesiology 123 (6), 1301–1311. 10.1097/ALN.0000000000000891 26492475 PMC4679549

[B16] Kulka P. J. Tryba M. Zenz M. (1996). Preoperative alpha2-adrenergic receptor agonists prevent the deterioration of renal function after cardiac surgery: Results of a randomized, controlled trial. Crit. Care Med. 24 (6), 947–952. 10.1097/00003246-199606000-00012 8681596

[B17] Lee S. (2019). Dexmedetomidine: Present and future directions. Korean J. Anesthesiol. 72 (4), 323–330. 10.4097/kja.19259 31220910 PMC6676029

[B18] Leino K. Hynynen M. Jalonen J. Salmenpera M. Scheinin H. Aantaa R. (2011). Renal effects of dexmedetomidine during coronary artery bypass surgery: A randomized placebo-controlled study. BMC Anesthesiol. 11, 9. 10.1186/1471-2253-11-9 21605394 PMC3123640

[B19] Liang H. Liu H. Z. Wang H. B. Zhong J. Y. Yang C. X. Zhang B. (2017). Dexmedetomidine protects against cisplatin-induced acute kidney injury in mice through regulating apoptosis and inflammation. Inflamm. Res. 66 (5), 399–411. 10.1007/s00011-017-1023-9 28224201

[B20] Liu X. Li Y. Kang L. Wang Q. (2021). Recent advances in the clinical value and potential of dexmedetomidine. J. Inflamm. Res. 14, 7507–7527. 10.2147/JIR.S346089 35002284 PMC8724687

[B21] Ljungqvist O. Scott M. Fearon K. C. (2017). Enhanced recovery after surgery: A Review. JAMA Surg. 152 (3), 292–298. 10.1001/jamasurg.2016.4952 28097305

[B22] Mathis M. R. Naik B. I. Freundlich R. E. Shanks A. M. Heung M. Kim M. (2020). Preoperative risk and the association between hypotension and postoperative acute kidney injury. Anesthesiology 132 (3), 461–475. 10.1097/aln.0000000000003063 31794513 PMC7015776

[B23] Meersch M. Schmidt C. Zarbock A. (2017). Perioperative acute kidney injury: An under-recognized problem. Anesth. Analg. 125 (4), 1223–1232. 10.1213/ANE.0000000000002369 28787339

[B24] Nadim M. K. Forni L. G. Bihorac A. Hobson C. Koyner J. L. Shaw A. (2018). Cardiac and vascular surgery-associated acute kidney injury: The 20th international consensus conference of the ADQI (acute disease quality initiative) group. J. Am. Heart Assoc. 7 (11), e008834. 10.1161/jaha.118.008834 29858368 PMC6015369

[B25] Park J. T. (2017). Postoperative acute kidney injury. Korean J. Anesthesiol. 70 (3), 258–266. 10.4097/kjae.2017.70.3.258 28580076 PMC5453887

[B26] Park S. W. Kim M. Kim J. Y. Ham A. Brown K. M. Mori-Akiyama Y. (2012). Paneth cell-mediated multiorgan dysfunction after acute kidney injury. J. Immunol. 189 (11), 5421–5433. 10.4049/jimmunol.1200581 23109723 PMC3504173

[B27] Patschan D. Müller G. A. (2016). Acute kidney injury in diabetes mellitus. Int. J. Nephrol. 2016, 6232909. 10.1155/2016/6232909 27974972 PMC5126418

[B28] Prowle J. R. Forni L. G. Bell M. Chew M. S. Edwards M. Grams M. E. (2021). Postoperative acute kidney injury in adult non-cardiac surgery: Joint consensus report of the acute disease quality initiative and PeriOperative quality initiative. Nat. Rev. Nephrol. 17 (9), 605–618. 10.1038/s41581-021-00418-2 33976395 PMC8367817

[B29] Schrier R. W. Wang W. (2004). Acute renal failure and sepsis. N. Engl. J. Med. 351 (2), 159–169. 10.1056/NEJMra032401 15247356

[B30] Shan X. S. Dai H. R. Zhao D. Yang B. W. Feng X. M. Liu H. (2021). Dexmedetomidine reduces acute kidney injury after endovascular aortic repair of stanford type B aortic dissection: A randomized, double-blind, placebo-controlled pilot study. J. Clin. Anesth. 75, 110498. 10.1016/j.jclinane.2021.110498 34488061

[B31] Shen J. Chu Y. Wang C. Yan S. (2022). Risk factors for acute kidney injury after major abdominal surgery in the elderly aged 75 years and above. BMC Nephrol. 23 (1), 224. 10.1186/s12882-022-02822-7 35739472 PMC9229523

[B32] Si Y. Bao H. Han L. Shi H. Zhang Y. Xu L. (2013). Dexmedetomidine protects against renal ischemia and reperfusion injury by inhibiting the JAK/STAT signaling activation. J. Transl. Med. 11, 141. 10.1186/1479-5876-11-141 23759023 PMC3700850

[B33] Taoda M. Adachi Y. U. Uchihashi Y. Watanabe K. Satoh T. Vizi E. S. (2001). Effect of dexmedetomidine on the release of [3H]-noradrenaline from rat kidney cortex slices: Characterization of alpha2-adrenoceptor. Neurochem. Int. 38 (4), 317–322. 10.1016/s0197-0186(00)00096-6 11137626

[B34] Vestergaard S. V. Christiansen C. F. Thomsen R. W. Birn H. Heide-Jørgensen U. (2021). Identification of patients with CKD in medical databases: A comparison of different algorithms. Clin. J. Am. Soc. Nephrol. 16 (4), 543–551. 10.2215/cjn.15691020 33707181 PMC8092062

[B35] Zappitelli M. Parikh C. R. Kaufman J. S. Go A. S. Kimmel P. L. Hsu C. Y. (2020). Acute kidney injury and risk of CKD and hypertension after pediatric cardiac surgery. Clin. J. Am. Soc. Nephrol. 15 (10), 1403–1412. 10.2215/cjn.00150120 32948644 PMC7536759

