# Supplementary material for: Correlation between perioperative dexmedetomidine administration and postoperative acute kidney injury in hypertensive patients undergoing non-cardiac surgery
Source: Front Pharmacol. 2023 Mar 29;14:1143176. doi: 10.3389/fphar.2023.1143176 (PMC10090366; doi:10.3389/fphar.2023.1143176)
Supplement: Supplementary file 1 [file DataSheet1.PDF]

Supplementary Table 1 The number of each surgery type included in this study

	without dexmedetomidine (n=5186)		with dexmedetomidine (n=583)	
	**Selective**	**Emergency**	**Selective**	**Emergency**
Gynecological surgery	659 (14.753%)	39 (5.424%)	94 (18.217%)	5 (7.463%)
Orthopedic surgery	1047 (23.439%)	117 (16.273%)	121 (23.450%)	15 (22.388%)
General surgery	1977 (44.258%)	321 (44.645%)	210 (40.698%)	23 (34.328%)
Neurosurgery	279 (6.246%)	157 (21.836%)	27 (5.233%)	14 (20.896%)
Thoracic surgery	251 (5.619%)	37 (5.146%)	37 (7.171%)	4 (5.970%)
Other surgery	254 (5.686%)	48 (6.676%)	27 (5.233%)	6 (8.955%)

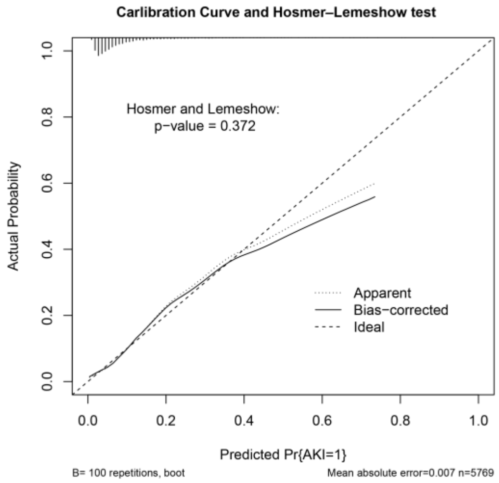

Supplementary Figure 1 Calibration curve and Hosmer-lemeshow test.
